# In-Patient Treatment of Fibromyalgia: A Controlled Nonrandomized Comparison of Conventional Medicine versus Integrative Medicine including Fasting Therapy

**DOI:** 10.1155/2013/908610

**Published:** 2013-01-23

**Authors:** Andreas Michalsen, Chenying Li, Katharina Kaiser, Rainer Lüdtke, Larissa Meier, Rainer Stange, Christian Kessler

**Affiliations:** ^1^Charité-University Medical Center, Institute of Social Medicine, Epidemiology and Health Economics, 10098 Berlin, Germany; ^2^Department of Internal and Complementary Medicine, Immanuel Hospital Berlin, 14109 Berlin, Germany; ^3^Karl und Veronica Carstens-Foundation, 45276 Essen, Germany

## Abstract

Fibromyalgia poses a challenge for therapy. Recent guidelines suggest that fibromyalgia should be treated within a multidisciplinary therapy approach. No data are available that evaluated multimodal treatment strategies of Integrative Medicine (IM). We conducted a controlled, nonrandomized pilot study that compared two inpatient treatment strategies, an IM approach that included fasting therapy and a conventional rheumatology (CM) approach. IM used fasting cure and Mind-Body-Medicine as specific methods. Of 48 included consecutive patients, 28 were treated with IM, 20 with CM. Primary outcome was change in the Fibromyalgia Impact Questionnaire (FIQ) score after the 2-week hospital stay. Secondary outcomes included scores of pain, depression, anxiety, and well being. Assessments were repeated after 12 weeks. At 2 weeks, there were significant improvements in the FIQ (*P* < 0.014) and for most of secondary outcomes for the IM group compared to the CM group. The beneficial effects for the IM approach were reduced after 12 weeks and no longer statistically significant with the exception of anxiety. Findings indicate that a multimodal IM treatment with fasting therapy might be superior to CM in the short term and not inferior in the mid term. Longer-term studies are warranted to assess the clinical impact of integrative multimodal treatment in fibromyalgia.

## 1. Introduction

Fibromyalgia is a complex clinical pain syndrome. Patients typically suffer from widespread musculoskeletal pain, fatigue, insomnia, and impairment of physical and psychological quality of life [1, 2]. The international prevalence of fibromyalgia ranges from 0.7 to 3.3% in the general adult population with an increase in recent years and a continuous majority of female patients [2–4].

The etiology of fibromyalgia is still unclear, although research showed an abnormal pain processing and lowered mechanical and thermal pain threshold by fMRI [5] as well as dysfunction of descending pain modulatory systems, for example, in the rostral anterior cingulate cortex (rACC) [6] and distinct neurotransmitter activities in cerebrospinal fluid [7]. Further discovered dysfunctions of the neuroendocrine axis could explain concomitant complaints as fatigue, irritable bowel, and mood disorders that are predominant in most of the fibromyalgia patients [8]. An association with psychosocial stressors is most likely [1, 8, 9].

Recent guidelines recommend a multimodal, multidisciplinary therapeutic approach involving medication, exercise, patient education, and behavioral and psychosomatic therapy [4, 10]. Due to frequent unsatisfying results of conventional treatment a substantial proportion of patients use complementary and integrative approaches such as Mind-body medicine, supplements, acupuncture, massage, and various nutritional therapies [11]. Clinical experience and preliminary evidence from uncontrolled prospective studies suggest that an integrative approach including nutritional and fasting therapies may help to decrease symptoms and increase the quality-of-life in inpatients with fibromyalgia [12, 13]. However, it would be useful to know how such an Integrative Medicine approach compares with conventional multimodal treatment which is established in specialized hospital units of rheumatology or pain medicine.

Prolonged modified fasting (Fasting cure, fasting therapy) with defined periods of voluntary abstention from solid food and a daily total energy intake <500 kcal has been found effective in several randomized trials on rheumatoid arthritis [14, 15]. The anti-inflammatory, pain relieving, antinociceptive, and mood-enhancing effects of fasting and caloric restriction have been well described in experimental and clinical studies [16–19]. Both, patients with rheumatoid arthritis and fibromyalgia frequently report that elimination diets and meal skipping alleviate their symptoms [13, 20, 21]. In a controlled nonrandomized study on the influence of a mediterranean diet or a fasting cure on the intestinal microflora the subgroup of patients with fibromyalgia experienced a greater improvement than nonfasters [13]. In another trial with a heterogeneous sample of chronic pain patients fasting led to an amelioration of mood and well-being [22].

In Germany, several academic hospital departments for naturopathic and integrative medicine have accumulated clinical experience in inpatient treatments of fibromyalgia. Within the treatment concepts of the integrative approach, modified fasting therapy is a mainstay of therapy. Notably, fasting treatments have been found to enhance health-promoting lifestyle modification [12], thus supporting a further key element of integrative therapy in fibromyalgia, mind-body medicine. 

We conducted this first controlled nonrandomized pilot-study to compare an integrative treatment strategy including fasting cure with a conventional rheumatologic treatment strategy.

We investigated quality of life, pain intensity, and psychological outcomes before and after the treatment of fibromyalgia in inpatients of two different departments of Internal Medicine, Integrative Medicine, and Rheumatology, of the same hospital, which is a tertiary center for Rheumatologic diseases. We hypothesized that fasting and integrative treatment would lead to a beneficial add-on effect with regards to quality-of-life, pain, and further psychological outcomes at time of hospital dismissal.

## 2. Material and Methods

### 2.1. Study Design and Participants

The study was conducted as a prospective, controlled nonrandomized study. The study protocol was reviewed and approved by the Ethics Committee of the Charité-University Medical Center, Berlin, and all patients gave their informed consent to study participation. Collection of data was performed by trained study personnel. 

All study subjects were inpatients from two departments of the Immanuel Hospital Berlin which is specialized in the treatment of rheumatic and chronic pain diseases, (1) patients of the Department of Integrative and Complementary Medicine and, (2) patients of the Department of Internal Medicine and Rheumatology. The primary diagnosis and reason for hospital admission of all participants was primary fibromyalgia. The study sample consisted of consecutively admitted inpatients during a 9-month period, who regularly stayed 14 ± 2 days in hospital for multidisciplinary treatment.

Inclusion criteria were a manifest fibromyalgia, as diagnosed by a rheumatologist, pain specialist, or internist, an age between 18 and 70 years, and a BMI between 20 and 45 kg/m². Patients with a start or change in drug therapy of their FMS less than 6 weeks ago, clinical relevant progressive or malignant diseases, current addiction or pregnancy, and inadequate cognitive abilities of cooperation were not included in the study. Further exclusion criteria were eating disorders, manifest liver disease, renal failure, gastric ulcer, and severe comorbidity including cancer and AIDS, premedication with immunosuppressive drugs (except corticosteroids) or coumarins, alcoholism, malnutrition, serious chronic infections, psychosis, epilepsy, type-1 diabetes, pregnancy, lactation, and a weight loss during the previous 3 months of >3 kg. 

### 2.2. Interventions

#### 2.2.1. Conventional Treatment

The conventional rheumatologic treatment approach consisted of a complex multidisciplinary treatment schedule with the following elements: group physiotherapy, hydrotherapy, thermal therapy, psychosomatic therapy, aerobic exercise, pool exercise, cognitive behavioral therapy, and education. The integrative and Complementary Medicine approach used the same treatment elements. In addition, fasting therapy and nutritional therapy supported by a group-based Mind-Body-Medicine concept was applied. The patients of both departments received a similar global amount of treatments with a total of 1600 to 2200 treatment minutes within the 2-week hospital period, according to agreements with health insurance companies in Germany. 

The method of fasting was adapted from the technique described by Buchinger [23–26]. A fasting period with 7 to 8 days of subtotal caloric restriction (daily nutritional energy intake <500 kcal) was predefined. Fasting was preceded by one or two prefasting days, using a 800 kcal/day monodiet of fruit, rice, or potatoes according to patients' choice. Fasting then began the following day with ingestion of an oral laxative, Natrium sulfuricum (“Glauber's salt”, 20–40 g). During fasting an enema or, if not wished by the patient, a mild laxative was applied every other day. The patients were recommended to drink 2-3 L of fluids each day (mineral water, small quantities of juice, and herbal teas). Vegetable broth was taken at lunch. The daily energy intake during the fast amounted to 350 kcal/day. For breaking the fast an apple was slowly eaten. The breakfast was followed by stepwise reintroduction of food with achievement of normocaloric intake by vegetarian meals on the third postfasting day. In the postfasting days a focus is set on reintroducing mindfulness to eating. 

Both departments are well experienced with the treatment of fibromyalgia syndrome and patients are received in a general appreciating manner. Inpatient treatments for fibromyalgia syndrome are recommended by German S-3 guidelines [1] and by health insurance companies for patients which do not respond adequately to outpatient care, including multimodal outpatient treatment. Patients are referred to both departments by internists, family practitioners, and rheumatologists comparably with patients' preference for Integrative Medicine and fasting treatment being the main criteria for choice of hospital department.

### 2.3. Measurements

All measures were assessed by trained study nurses at three study visits, at baseline, after 2 weeks (at dismissal from hospital) and at study week 12 (10 weeks after dismissal). The primary outcome measure was the change in the Fibromyalgia Impact Questionnaire (FIQ) score from baseline to the end of the in-hospital intervention. The FIQ is a validated, multidimensional measure to assess the severity of fibromyalgia as rated by patients. The total score ranges from 0 to 100, with higher scores indicating more severe symptoms [27]. The validated German version was used [28].

Global pain status was assessed additionally by asking the patients for the global severity of the disease-related pain by means of a self-rating 100 mm Visual Analogue Scale (VAS) with a value of 100 indicating maximum pain and 0 indicating no pain. Patients were carefully instructed before first self-ratings on the correct use of the VAS.

Prespecified other secondary outcomes included (1) a 100 mm visual analogue scale for self-rated global quality of sleep; (2) the German version of the Spielberger State-Trait Anxiety Inventory (STAI), which consists of 20 items relating to state anxiety and 20 items relating to trait anxiety [29]; (3) the Bf-S Zerssen well-being scale, which measures momentary emotional well-being and consist of three answer categories with higher scores indicating lower well-being [30]; (4) the German version of the Hospital Anxiety and Depression Scale (HADS) [31], a validated standard measure for anxiety and depression which uses a 14-item scale with seven of the items related to anxiety and seven related to depression [32]; (5) the German version of the Pain Perception Scale for Adolescents (SES), which assesses sensory pain perception in chronic pain patients [33]. 

Subjects height and body weight were measured following a standardized protocol while patients wore light clothing and no shoes after an overnight fast. BMI was calculated as weight (kg)/height^2^ (m). Anthropometrical and clinical data were collected by trained study personnel. Seated blood pressure was measured after 5 min rest with a calibrated sphygmomanometer at the nondominant arm by trained nurses.

### 2.4. Statistical Analysis

As the study was designed as a nonrandomized pilot study no sample size calculation was conducted. However, we intended to include 60 patients and assumed a drop-out rate of 15%, giving a study sample of about 50 patients with full data sets. 

Baseline differences were calculated by Kruskal-Wallis test. All outcome criteria were analyzed by intention-to-treat; including all subjects, irrespective whether or not they adhered to the protocol or gave a full set of data. For each outcome we fitted a generalized estimation equation (GEE), analysis of covariance (ANCOVA) which included treatment group (binary covariate), and the respective baseline value (linear covariable) as independent variables. Treatment effects were estimated within these models, and reported as adjusted group differences including their respective 95% confidence intervals (CI) and *P* values. All reported *P* values were based on two-sided tests, and a *P*-value < 0.05 was considered significant. All statistical computations were performed with SAS/STAT statistical software version 9.1 (SAS institute, Cary, North Carolina, USA).

## 3. Results 

### 3.1. Baseline

During the 9-month study recruitment period we screened 56 screened patients with manifest fibromyalgia which were admitted to one of the two hospital departments. Of these, 48 volunteered to participate in our study; 20 in the department of Rheumatology and 28 in the department of Integrative and Complementary Medicine. Data assessments were complete for study visits 1 (baseline) and 2 (week 2). After 12 weeks data from 25 patients of the department of Integrative and Complementary Medicine and 17 of the department of Rheumatology were available. 

Baseline characteristics of the study population revealed a middle-aged and predominantly female study population. Patients of the Department of Rheumatology showed a significantly greater impaired quality of life, the primary outcome, and had slightly higher pain scores and were more emotionally distressed with slightly higher scores for depression and anxiety compared to patients of the Department for Integrative and Complementary Medicine (Table 1). Use of medication prior and during the hospital stay, for example, with amitriptyline and other antidepressants, was not different between groups. 

### 3.2. Primary Outcome

The FIQ score decreased substantially in the Integrative Medicine Group and to a significantly greater extent compared to the Rheumatologic group after 2 weeks (Table 2). At 12 weeks, the FIQ score increased again in both groups resulting in improvements of only 12% for the integrative and fasting approach and 6% for the control group, resulting in a nonsignificant difference between the groups. 

### 3.3. Secondary Outcomes

At 2 weeks, the Integrative Medicine group had greater mean improvements in all secondary outcomes and most pronounced in the scores of quality of sleep, pain, pain perception, and anxiety (HADS, STAI) (Table 2). 

At 12 weeks, the pain score and pain perception score only showed a trend towards a beneficial outcome for the Integrative Medicine group compared to the Rheumatology group. All psychological outcomes were better in the Integrative Medicine group compared to the Rheumatologic group, however group differences were reduced and no longer statistically significant with the exception of anxiety. All of the outcomes deteriorated again compared to the 2-weeks data resulting in mild mid-term treatment effects compared to baseline levels. 

### 3.4. Safety

There were no serious adverse events in both groups. About 35% in each group reported some minor side effect. Within the Integrative Medicine group the first fasting days were frequently accompanied by dizziness, minor headache, and tiredness. Patients in the Rheumatology group reported frequently about muscle pain and tiredness, most likely due to exercise and physical therapies. 24 out of 28 patients in the integrative Medicine group declared that they would participate in fasting as again. 17 out of 20 patients in the Rheumatology group declared that they would like to repeat the treatment. 

## 4. Discussion 

In this controlled nonrandomized trial we compared the effectiveness of two time- and attention-balanced inpatient multimodal treatment strategies: an Integrative Medicine approach that included fasting therapy versus the conventional Rheumatologic therapy. While patients in the Rheumatologic group were more diseased at baseline, adjusted data analysis showed a more beneficial effect of the Integrative Medicine approach after 2 weeks for all of the clinical outcomes. At week 12, effects in both groups were reduced but still favored the Integrative Medicine approach, for example, for the psychological outcomes. The minimally clinically important difference of the FIQ is estimated to amount to 14%. In the present study the reduction of the FIQ at 2 and 12 weeks was 30.2% and 12.2% with Integrative Medicine versus 13.1% and 6.0% with multimodal Rheumatologic care. Thus, our results point to a relevant immediate effect of the Integrative Medicine approach while the long-term effects appear to be only mild. 

We were surprised to see an only mild effectiveness of the Rheumatologic multimodal treatment approach although it combined several evidence-based treatment methods such as aerobic exercise, pool exercise, thermal therapy, psychotherapy, and cognitive behavioral therapy. However, it has to be noted that patients that are admitted to an inpatient treatment in Germany are highly selected as they have to be documented nonresponders to outpatient treatments according to requirements of health insurance companies and thus may be especially difficult to treat. 

A recent study has evaluated the effects of a conventional multimodal inpatient treatment of 3 weeks within the setting of a specialized Rheumatologic rehabilitation hospital [34]. For the outcomes that were used (Pain, HADS) the results of the Integrative Medicine approach used in this study were also favorable, thus confirming our results.

Principally, treatment of fibromyalgia is still unsatisfying and most patients continue to be in considerable pain years after the first diagnosis and experience reduced quality of life. New approaches are needed and the majority of patients with fibromyalgia frequently also use methods of complementary medicine. Various types of exercise and mind-body medicine have been advocated, yet long-term adherence is limited. In Germany, nutritional therapies and fasting are very popular. Fasting treatments have found to be effective in the treatment of rheumatoid arthritis and pain syndromes, furthermore they may support motivation and self-efficacy in health-promoting lifestyle modification [12, 15, 21, 35]. In a preliminary study we observed a moderate pain-relieving effect of fasting in fibromyalgia [13]. 

Of note, we found a partially persisting mood-enhancing effect in the integrative medicine group which may be related to fasting therapy. Previous research has documented mood-enhancing effects of caloric restriction and fasting. Several mechanisms including increased central serotonin availability have been described experimentally [17]. 

Only a few studies have investigated multimodal treatment programs for fibromyalgia that focus on Integrative and Complementary Medicine. A small uncontrolled study in 28 patients found an Ayurvedic program, also focusing on nutrition and mind-boy techniques, to be effective with a lasting effect up to 24 months [36]. However, the treatment was not compared to another intervention, thus selection bias and unspecific effects were most likely contributing factors to the effect. 

In view of our documented effects and safety of the Integrative Medicine approach further research on the effectiveness of complex multimodal Integrative treatments and comparisons with standard care in fibromyalgia is warranted. Such a study should have a larger sample size, allocate patients randomly, and include an attention control for the fasting intervention. Here the conventional group could be deprived of some specific food ingredient without inducing fasting metabolism. As it is difficult to randomize patients into complete treatment settings due to patient preferences and obligations of cost coverage, also outcome research might be useful in benchmarking the best strategy in intensified treatment strategies of fibromyalgia.

 Some limitations relate to our study. First, we used a nonrandomized study design as it is currently not possible to randomize patients to hospital departments when costs are covered by health insurance companies under usual care. Nonrandomized studies may introduce a bias by patient selection and different prognostic and response factors between the groups. In fact, baseline values found patients of the Rheumatologic department to be more diseased and more distressed. However, most of the baseline differences were statistically nonsignificant and all our data analysis included baseline values as covariates. Of note, Physicians can refer patients to both hospital departments only if they are documented nonresponders to intensive outpatient outpatient treatment. The selection of the department (Rheumatology or Integrative Medicine) is mainly influenced by patients' preference. Here a specific selection bias may be introduced as patients interested in integrative Medicine are possibly more likely to search for comprehensive treatments in less severe disease states. Second, our study population was of limited size. Smaller study populations hold the risk of overestimation of effects on the one side and nondetection of moderate treatment effects on the other side. However, if significant effects are found the magnitude of effects and the related possible clinical relevance of the intervention is emphasized, which our results reflect. A third limitation is the short observation period of 3 months. Further studies should include observation periods of 12 months and longer to assess long-term symptom control. 

A strength of our study relates to the fact, that both departments are situated in the same hospital and that, beside fasting and mind-body medicine, all other treatments were comparable and applied by the same personnel. Thus setting effects, attention effects and other nonspecific factors that may otherwise introduce bias in comparative studies were minimized.

In conclusion, our preliminary findings indicate that a multimodal Integrative Medicine treatment approach that included fasting therapy might be superior to the multimodal conventional Rheumatologic approach in the short-term in patients with severe fibromyalgia. At 12 weeks neither of the studied interventions was significantly superior or achieved clinically relevant improvement. Longer-term studies are warranted to assess the clinical impact and potential of multimodal Integrative Medicine in fibromyalgia.

## Figures and Tables

**Table 1:** Baseline characteristics.

Characteristics	Integrative medicine group	Rheumatology group	*P* value
Male/Female, No.	0/28	2/18	
Age, years	53.6 ± 10.8	51.8 ± 10.1	0.516
Body mass index, kg/m^2^	27.8 ± 4.5	30.3 ± 6.7	0.281
SBP, mm Hg	122.3 ± 13.2	128.5 ± 13.7	0.072
DBP, mm Hg	76.8 ± 7.8	78.8 ± 10.1	0.656
Physical well-being	7.1 ± 1.9	8.0 ± 1.2	0.089
Practice of exercise, No. /(%)	21 (75.0%)	13 (65.0%)	0.452
Practice of Relaxation, No. /(%)	8 (28.5%)	5 (25.0%)	0.784
FIQ score	54.3 ± 15.0	68.0 ± 8.9	0.004
Pain score	58.2 ± 19.6	66.5 ± 19.5	0.135
Quality of sleep	60.5 ± 26.7	68.3 ± 27.6	0.191
STAI state score	50.4 ± 11.2	58.4 ± 13.0	0.027
STAI trait score	51.1 ± 11.2	54.3 ± 12.0	0.341
HADS-Anxiety	10.4 ± 3.8	11.3 ± 5.1	0.607
HADS-Depression	8.3 ± 4.8	11.1 ± 5.2	0.055

Values are mean ± SD if not indicated otherwise. SBP: systolic blood pressure; DBP: diastolic blood pressure.

STAI: State and Trait Anxiety questionnaire, FIQ: Fibromyalgia impact questionnaire; HADS: Hospital Anxiety and Depression scale.

**Table 2:** Outcomes in both groups at baseline, week 2 and 12 with group differences as indicators of change.

	Integrative medicine group			Rheumatology group			Mean diff		Mean diff (95% CI)	
	Baseline	Visit 2	Visit 3	Baseline	Visit 2	Visit 3	∇ 1-2 (95% CI)	*P* value	∇ 1–3 (95% CI)	*P* Value
FIQ score	54.3 ± 15.0	38.0 ± 17.3	47.7 ± 19.3	68.0 ± 8.9	59.1 ± 15.3	63.9 ± 20.7	−11.2 (−20.1, −2.3)	0.014	−6.7 (−17.5, 4.1)	0.223
Pain score	58.2 ± 19.6	37.4 ± 19.9	48.8 ± 26.1	66.5 ± 19.5	58.3 ± 22.8	64.4 ± 25.7	−17.5 (−28.8, −6.1)	0.003	−13.0 (−28.0, 2.1)	0.091
Quality of sleep	60.5 ± 26.7	43.6 ± 27.8	48.3 ± 26.4	68.3 ± 27.6	61.1 ± 29.0	67.4 ± 20.4	−15.5 (−30.6, −0.4)	0.044	16.5 (−30.0, −3.1)	0.016
HADS Anxiety	10.4 ± 3.8	6.9 ± 3.2	8.9 ± 3.9	11.3 ± 5.1	10.3 ± 5.0	11.6 ± 4.2	−2.9 (−4.6, −1.3)	<.001	−2.3 (−3.8, −0.9)	0.002
HADS Depression	8.3 ± 4.8	6.1 ± 4.2	7.8 ± 4.1	11.1 ± 5.2	9.3 ± 5.1	11.6 ± 4.2	−1.5 (−3.3, 0.3)	0.097	−2.0 (−4.0, −0.0)	0.046
SES Pain Perception	30.7 ± 9.1	22.1 ± 6.6	29.8 ± 10.2	36.4 ± 8.6	34.2 ± 9.1	37.0 ± 10.5	−10.1 (−14.7, −5.5)	<.001	−5.2 (−11.0, 0.5)	0.075
STAI State Score	50.4 ± 11.2	39.7 ± 10.2	48.2 ± 11.5	58.4 ± 13.0	51.4 ± 12.6	57.6 ± 11.5	−8.2 (−13.9, −2.4)	0.005	−6.1 (−11.8, −0.4)	0.036
STAI Trait Score	51.1 ± 11.2	43.9 ± 9.7	48.6 ± 9.3	54.3 ± 12.0	53.5 ± 10.0	55.5 ± 9.6	−8.3 (−12.3, −4.2)	<.001	−4.8 (−8.9, −0.7)	0.022
BfS Well-being	21.3 ± 11.6	17.5 ± 14.1	23.4 ± 11.8	27.2 ± 9.0	27.8 ± 8.2	26.0 ± 10.4	−6.8 (−12.5, −1.2)	0.018	−1.4 (−8.0, 5.2)	0.675

Values are mean ± SD if not indicated otherwise. SBP: systolic blood pressure; DBP: diastolic blood pressure.

STAI: State and Trait Anxiety questionnaire, FIQ: Fibromyalgia impact questionnaire; HADS: Hospital Anxiety and Depression scale;

∇ 1-2 = difference between groups from baseline to visit 2 at 2 weeks, ∇ 1–3 = difference between groups from baseline to visit 3 at 12 weeks,

*P values for between group difference of change, adjusted.
